# Flash Sintering of YSZ/Al_2_O_3_ Composites: Effect of Processing and Testing Conditions

**DOI:** 10.3390/ma14041031

**Published:** 2021-02-22

**Authors:** Mattia Biesuz, Andrea Ometto, Vincenzo Maria Sglavo

**Affiliations:** Department of Industrial Engineering, University of Trento, Via Sommarive 9, 38123 Trento, Italy; andreaometto4@gmail.com (A.O.); vincenzo.sglavo@unitn.it (V.M.S.)

**Keywords:** flash sintering, ceramic composites, alumina, YSZ, field-assisted sintering

## Abstract

The flash sintering behavior of yttria-stabilized zirconia/alumina composites was investigated to understand the role of the fundamental processing and testing parameters (electric field intensity, electric current limit, thermal insulation, homogeneity and dispersion of the two phases) on densification. A strong relation between the composite compositions and the electric parameters needed to promote flash sintering is revealed. Interestingly, the composite preparation method, which affects the two-phases dispersion homogeneity, was shown to have a relevant effect on the flash onset conditions, where the more homogeneous material is more difficult to be flashed. Moreover, the use of a simple thermal insulation system around the green body allowed to improve the final density of the composites under constant electric current.

## 1. Introduction

Flash sintering (FS) is a novel field-assisted sintering technology [1,2,3,4] where an electric field is directly applied to a ceramic green body while it is heated up within a conventional furnace. An electric current is forced to pass through the ceramic activating, at an onset combination of electric field and furnace temperature, the so called flash event [4,5,6] which induces Joule heating and ultra-rapid sintering. The flash transition is also accompanied by a bright glowing and a drop in the electrical resistivity which are considered a footprint of the process [4,5,6]. Such amazing effect, which changes the sintering time form 10^4^ s to 10^1^ s range, has attracted a relevant scientific interest aiming at unraveling the physical and chemical mechanisms behind the phenomenon [7,8,9,10,11,12,13,14,15,16,17,18,19,20,21,22]. Although definitive conclusions cannot be drawn yet, the mainstream is that the process is predominantly driven by the Joule effect coupled with side field/current-induced *athermal* phenomena [23] which can be possibly use to develop ceramics with new functional [24,25,26,27] and mechanical [28] properties. On the other hand, several researchers focused on the application of the process to different class of ceramics employing commercially-available spark plasma sintering [26,29,30,31,32] and microwave sintering [33,34] equipment.

Since flash sintering is an electrically-activated phenomenon, a certain interest has risen in the consolidation of composites containing phases characterized by different electrical properties [35,36,37,38,39,40,41,42,43], where the conductive one facilitates the flash activation. Yttria-stabilized zirconia (YSZ)/alumina composites are among the most studied compositions; in fact, they are model systems combining a good ionic conductor and an insulator. Over than the scientific relevance of the composite in the field of flash sintering, biphasic YSZ / Al_2_O_3_ systems are among the most diffuse technical ceramics for structural application as they combine relatively lightweight and low price (Al_2_O_3_) with high fracture toughness (YSZ) and refractory properties. The first studies were carried out by Naik et al., who investigated the onset conditions and grain coarsening upon FS in 50 vol.% YSZ/Al_2_O_3_ composite [40]. More recently, Ojaimi et al., have studied the densification of 75 vol.% ZrO_2_/25 vol.% Al_2_O_3_ composite using two different current and voltage limits [39]. Finally, M’Peko investigated the onset flash condition in well-dispersed YSZ/Al_2_O_3_ composites with different compositions, pointing out that FS could not be activated if the YSZ content was lower than 30 vol.%. This was due to the lack of a percolative conductive phase if the YSZ load was below a threshold value.

The goal of the present work was to further investigate the flash sintering behavior of YSZ/Al_2_O_3_ composites and identify the role of composition, phases dispersion, electric current and electric field on densification which could be improved also by the use of a simple thermal insulation systems to avoid heat dissipation [44,45].

## 2. Materials and Methods

Commercially available high purity α-Al_2_O_3_ (Taimicron TM-DAR, Taimei Chemicals Co., Ltd., Tokyo, Japan, purity > 99.99%, SSA = 15.6 m^2^/g) and yttria-stabilized zirconia (TZ-8YSZ, TosohCorp., Shunan, Japan, SSA = 14.5 m^2^/g) were used in the present study. The two powders were mixed in an agate mortar with distilled water and 6 wt.% binder (B-1000, Duramax, DowChemical, Midland, MI, USA) in different volumetric composition (YSZ:Al_2_O_3_): 100:0, 50:50, 25:75, 15:85 and 10:90. The slurry was let to dry overnight at 80 °C.

An additional powder batch was produced by mixing 85 vol.% α-Al_2_O_3_ and 15 vol.% YSZ in toluene (Honeywell, 34494, Charlotte, NC, USA) overnight in a TURBULA^®^T2F (Artisan technology group, Champaign, IL, USA) high energy planetary mixer (together with zirconia balls) to obtain a more homogeneous dispersion of the two phases. The solvent was then evaporated in a fume hood. In the followings, this batch of sample is referred as “turbula mixed”.

Dog-bone samples were produced by uniaxial pressing at 120 MPa and, by average, the specimens’ cross section was 2.6 mm × 2.9 mm. The pressed green bodies were pre-sintered at 1100 °C for 1 h (heating rate = 10 °C/min). The density after pre-sintering was 49–50% of the theoretical one.

Two sets of FS experiments were carried out. The first one was performed to determine the onset temperature for the flash event. Flash sintering was accomplished at constant heating rate (20 °C/min) in a Linseis L75 PT (Linseis, Selb, Germany) horizontal dilatometer. Pt electrodes were inserted in the holes at the sample terminals, where Pt paste was previously placed. The electrodes were connected to a DC power supply (Glassman, 5 kV-120 mA) and a digital multimeter Keithley 2100 (Keithley, Solon, OH, USA). Once the system reached the current limit (set at 8 mA/mm^2^), the power source worked under current control for 120 s for all samples. The flash onset temperature was determined by the usual tangent method in the Arrhenius power dissipation plots (ln(power) vs. reciprocal of the absolute furnace temperature): the tangent to the power plot was determined before and after the non-linearity of the flash event, their intersection identifies the flash onset furnace temperature. 

The second set of experiments aimed at understanding the electric current effect on sintering. The samples were introduced in a pre-heated Nabertherm P330 (Carbolite Gero, Hope, UK) tubular furnace at 1000 °C and, after 5 min, the electric field was applied. Since composites with far different electric properties were tested, two power supplies were used Glassman 5 kV-120 mA (Glassman, Hauppauge, NY, USA); and Sorensen DLM 300-2 (AMETEK Programmable Power, San Diego, CA, USA). Current limits in the range 4–130 mA/mm^2^ were applied to the specimens and the set voltage limit was high enough to guarantee that the system worked in current control for the entire experiment. The samples were kept in the flash state for 120 s and then the power source was switched off. In order to understand the effect of a thermal insulation systems on the densification by FS, a 5 mm thick silicon nitride felt (produced according to [46]) was wrapped around some samples (50 vol.% of YSZ) before the test. All flash experiments were carried out in air.

The density of the sintered specimens was determined by the Archimedes’ method using an analytical balance (Gibertini) with sensitivity of 0.1 mg. The microstructure was studied by SEM (Jeol JSM 5500, Tokyo, Japan) on fresh fracture surfaces, the samples being coated with a thin Pt/Pd alloy layer by sputtering before the analysis.

## 3. Results and Discussion

**Figure 1:**
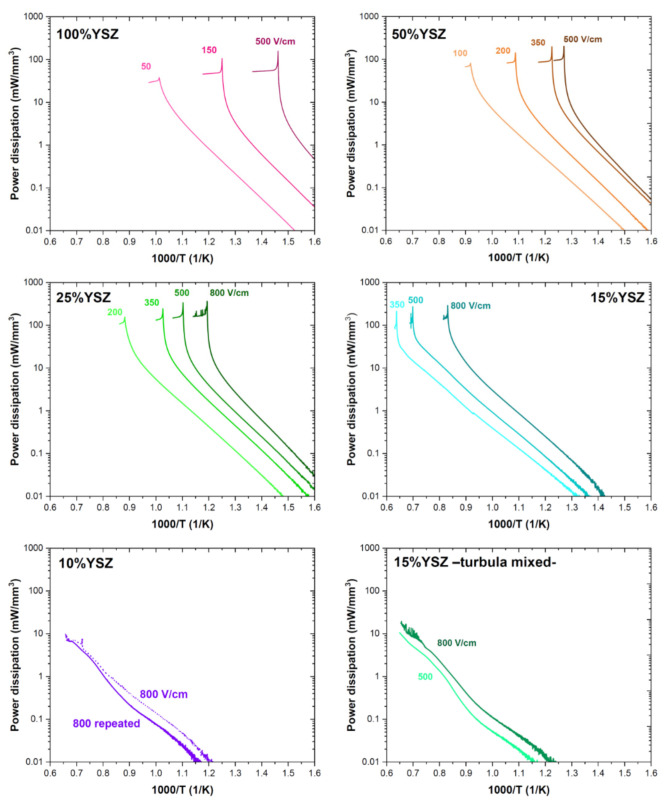
Specific power density as a function of the reciprocal of the furnace temperature for composites possessing different compositions (constant heating rate = 20 °C/min). Where not otherwise specified, the composites were produced by manually mixing YSZ and alumina powders. The sample containing 10 vol.% YSZ was tested twice under 800 V/cm to confirm the absence of the flash transition. All the plots refer to samples without thermal insulation, the current limit was 8 mA/mm^2^.

The power dissipation plots recorded during constant heating rate flash experiments are reported in Figure 1. The flash event is signaled by a non-linear power surge whose onset temperature depends on the applied electric field and on the composite composition. Interestingly, all composites produced by manually mixing the powders and containing 15 vol.% or more YSZ were flashed, whereas the samples containing only 10 vol.% conductive phase did not flash even using very high electric fields (800 V/cm). The flash sinterability of the composite containing 15 vol.% YSZ is a rather surprising result if one considers that 15 vol.% is theoretically below the percolation limit of the conductive phase, i.e., YSZ. In this regard, M’Peko [41] reported that well-dispersed YSZ/Al_2_O_3_ composites containing 20 vol.% (or less) YSZ cannot be flashed. To check our results a flash experiment was carried out on 15 vol.% YSZ/85 vol.% Al_2_O_3_ mixture produced by milling the ceramic powders in toluene using a Turbula mixer. Such processing route allowed to manufacture a homogeneous dispersion of the two phases. Figure 1 points out that the well-dispersed composite cannot be flash sintered under 500 V/m or 800 V/cm, whereas the same composition produced by manually mixing the powders was flashed with electric fields as low as to 350 V/cm. These results point out the relevance of the powder mixing method on the flash sintering behavior of the composites.

The slope of the log(power) vs. 1000/*T* plot during the flash incubation represents the activation energy for conductivity, *Q*. It is similar for all tested compositions ranging between 1.05 and 1.25 eV (Figure 1) and it is in rather good agreement with that measured for ionic conductivity in YSZ (typically reported around 0.9–1.1 eV [47]). The independency of *Q* on the composite composition suggests that the conduction mechanism is likely the same in all materials and it is related to the motion of oxygen ions in the percolative YSZ phase. It is worth spotting that, whereas conductivity is likely predominantly ionic during the incubation, it is expected to switch to electronic *n-type* at the flash onset [48,49] because of field-induced electrochemical reduction of YSZ under DC.

**Figure 2:**
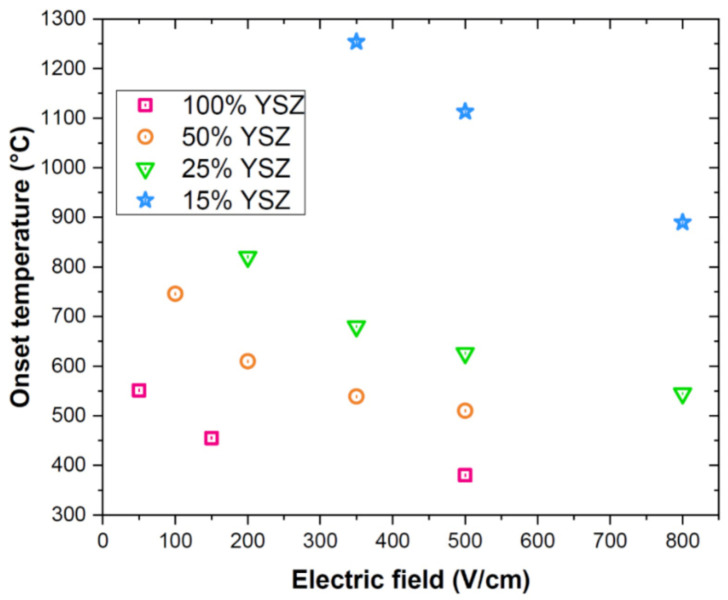
Flash onset furnace temperature as a function of the electric field and composite composition in samples produced by manually mixing the powders (constant heating rate = 20 °C/min). The samples were not thermally insulated and the current limit was 8 mA/mm^2^.

Figure 2 shows the effect of the composite composition and electric field on the flash onset temperature for materials produced by manually mixing the powders. As expected the onset flash temperature decreases when increasing the applied electric field [50,51] because of the quadratic dependence of the electric power dissipation on field strength. On the other hand, the composite composition plays a key role on the flash activation. In fact, the higher is the YSZ load, the lower is the flash onset temperature. This is clearly due to the fact that charge transport and power dissipation are possible only in the YSZ phase, high purity Al_2_O_3_ being a perfect insulator at the tested temperatures [52]. Therefore, as YSZ content increases, so does the composite electrical conductivity which facilitates the activation of the flash thermal runaway. The effect of the composite composition on the electrical conductivity is well-visible in the power dissipation plots (Figure 1) which shift upward with YSZ load. One can also observe that the flash onset temperature is non-linearly correlated with the composite composition. As an example, if samples treated with 500 V/cm are considered, one can observe that the FS temperature increases by ≈130 °C when YSZ is reduced from 100% to 50%. A further YSZ decrease by 25% produces an increase in the onset temperature by ≈110 °C and an additional reduction of 10% (from 25% to 15% YSZ) causes a surge of the flash temperature by nearly 500 °C. This suggests that the sample containing 15 vol.% YSZ is very close to the percolation limit of the conductive phase, below which flash sintering is not possible.

**Figure 3:**
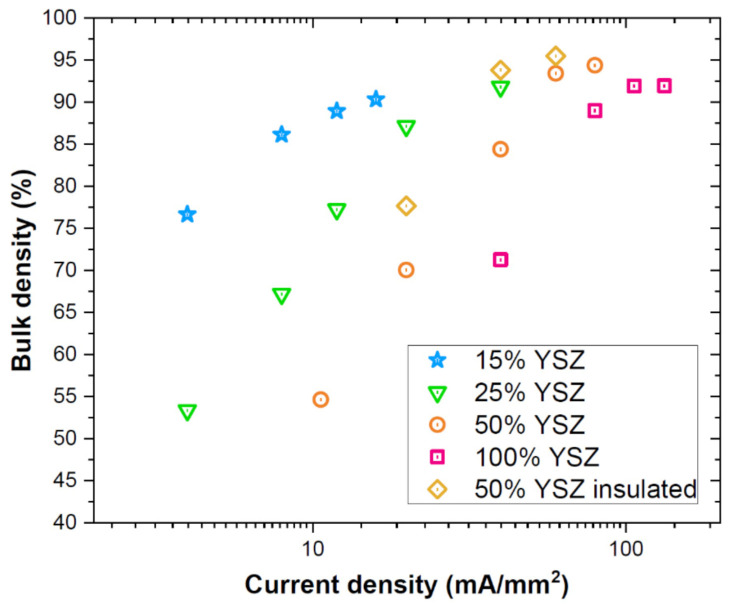
Bulk density of the flash sintered bodies as a function of the applied current density and composite composition (furnace temperature = 1000 °C, dwell time in the flash state = 120 s). The materials were produced by manually mixing the powders. Where not otherwise specified the samples were not thermally insulated. The power source was kept in current limit for whole the process.

The effect of the current limit on the ceramic bodies densification is reported in Figure 3. The results refer to the experiments carried out at constant furnace temperature (1000 °C) and maintaining the power source in current limit for the entire process (120 s), thus avoiding the incubation step for FS. One can observe high density (90%) can be achieved in all tested compositions. The relative density of all composites grows with the set current limit since Joule heating increases the sample temperature. The composition-related variation of the electrical conductivity causes a substantial shift in the current density required for densification: the larger is the YSZ load, the higher are the conductivity and the current required for densification. In particular, one can observe that the composite containing 15 vol.% YSZ can be densified with current limits in the order of 10^1^ mA/mm^2^, whereas pure YSZ requires currents in the order of 10^2^ mA/mm^2^. It is also worth mentioning that the relative density of both the turbula-mixed samples (which did not show the non-linearity of FS in the power plots, Figure 1) was only 50–52%. These values are only slightly above the pre-sintered materials density (49–50%), confirming that no substantial densification occurred. The result agrees with the idea that the flash is a power-activated phenomenon and only modest densification takes place during the incubation stage. Hence, the field alone does not appear to be the real reason for fast densification upon FS, the process being instead related to power dissipation.

**Figure 4:**
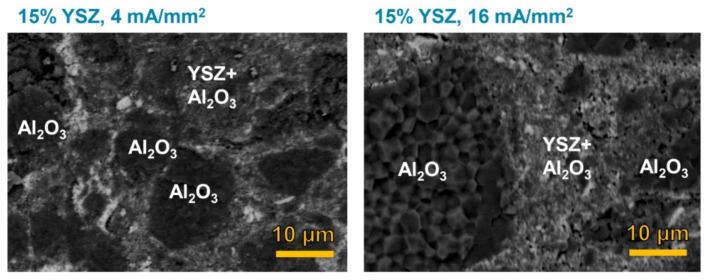
SEM micrographs of the composite containing 15 vol.% YSZ flashed with different current densities (furnace temperature = 1000 °C, dwell time in the flash state = 120 s). The samples were not thermally insulated and the power source was kept in current limit for the entire process. The materials were produced by manually mixing the powders.

The SEM micrographs in Figure 4 confirm the current effect on densification, pointing out that a current limit increase improves densification and activates grain coarsening. One can also notice that the composites manufactured by manually mixing the powders (Figure 4) are not really homogeneous, they presenting big alumina islands within a mixed YSZ/Al_2_O_3_ matrix. The confinement of part of the insulating phase (i.e., Al_2_O_3_) within these islands decreases its load in the matrix where the electric current is supposed to flow. For such reason, it was possible to flash sinter composites containing only 15 vol.% YSZ, the real YSZ load in the percolative matrix being definitively larger than 15 vol.%. In this case, inhomogeneity helps.

A second key observation from Figure 4 is that the Al_2_O_3_ islands are fully dense and possess coarse grains although no current is supposed to pass through them. Therefore, their densification appears substantially related to thermal effects (not a current-induced phenomenon) although occurring in a few seconds. Although this can be considered a side result of the present work and additional investigations are probably required, it could represent a new approach to discriminate the current effects on sintering in the next future.

**Figure 5:**
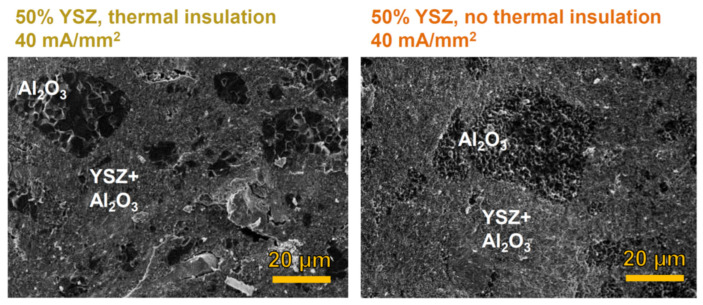
SEM micrographs of the composite containing 50 vol.% of YSZ and flash sintered with and without thermal insulation (furnace temperature = 1000 °C, dwell time in the flash state = 120 s). The power source was kept in current limit for the entire process. The materials were produced by manually mixing the powders.

Density measurements and SEM micrographs in Figure 3 and Figure 5 reveal that the use of ceramic felts to insulate the specimen (and limit heat dissipation) accounts for a significant FS improvement. In fact, the use of thermal insulation allows to increase the relative density by about 10% in the samples treated with 22 and 40 mA/mm^2^ (50 vol.% of YSZ). Moreover, the microstructure appears coarser because of the heat concentration within the sample. This is really evident in the alumina islands, where the grain size exploded when employing thermal insulation. This further confirms the predominant thermal nature of the mass transport phenomena in the alumina pockets.

## 4. Conclusions

Yttria-stabilized zirconia/alumina composites can be densified by flash sintering if the conductive phase load exceeds 15 vol.%. The composite preparation method affects the flash onset conditions. In particular, the presence of Al_2_O_3_ agglomerates facilitate the activation of the flash transition even in mixtures containing only small amounts of zirconia.

The flash onset temperature strongly depends on the composite composition and on the applied electric field, whereas the density of the sintered bodies varies according to their composition and the current limit. Specifically, mixtures containing larger amount of YSZ require lower fields to be flashed and higher currents to be densified. Moreover, the densification is improved if the ceramic body is thermally insulated upon flash sintering.

## Data Availability

The data presented in this study are openly available in [Mendeley Data] at [http://dx.doi.org/10.17632/x6dgwwwjcf.1], reference [Flash sintering YSZ/alumina composites].
